# GWAS revealed a novel resistance locus on chromosome 4D for the quarantine disease Karnal bunt in diverse wheat pre-breeding germplasm

**DOI:** 10.1038/s41598-020-62711-7

**Published:** 2020-04-07

**Authors:** Sukhwinder Singh, D. Sehgal, S. Kumar, M. A. R. Arif, P. Vikram, C. P. Sansaloni, G. Fuentes-Dávila, C. Ortiz

**Affiliations:** 1 International Maize and Wheat Improvement Center (CIMMYT), Carretera México-Veracruz Km. 45, El Batán, Texcoco, C.P. 56237 México; 2 Geneshifters, 222 Mary Jena Lane, Pullman, WA 99163 USA; 3 Centre of Excellence in Biotechnology, Anand Agricultural University (AAU), Anand, Gujarat 388 110 India; 4 Nuclear Institute for Agriculture and Biology, Faislabad, 38000 Pakistan; 5 INIFAP-CIRNO, Campo Experimental Norman E. Borlaug, Apdo. Postal 155, Km 12 Norman E. Borlaug, Cd. Obregon, Sonora, CP 85000 Mexico

**Keywords:** Biotechnology, Genetics, Plant sciences

## Abstract

This study was initiated to identify genomic regions conferring resistance to Karnal Bunt (KB) disease in wheat through a genome-wide association study (GWAS) on a set of 179 pre-breeding lines (PBLs). A GWAS of 6,382 high-quality DArTseq SNPs revealed 15 significant SNPs (*P*-value <10^−3^) on chromosomes 2D, 3B, 4D and 7B that were associated with KB resistance in individual years. In particular, two SNPs (chromosome 4D) had the maximum *R*^2^ values: SNP 1114200 | F | 0–63:T > C at 1.571 cM and *R*^2^ of 12.49% and SNP 1103052 | F | 0–61:C > A at 1.574 cM and *R*^2^ of 9.02%. These two SNPs displayed strong linkage disequilibrium (LD). An *in silico* analysis of SNPs on chromosome 4D identified two candidate gene hits, TraesCS4D02G352200 (*TaNox8*; an NADPH oxidase) and TraesCS4D02G350300 (a rhomboid-like protein belonging to family S54), with SNPs 1103052 | F | 0–61:C > A and 1101835 | F | 0–5:C > A, respectively, both of which function in biotic stress tolerance. The epistatic interaction analysis revealed significant interactions among 4D and 7B loci. A pedigree analysis of confirmed resistant PBLs revealed that *Aegilops* species is one of the parents and contributed the D genome in these resistant PBLs. These identified lines can be crossed with any elite cultivar across the globe to incorporate novel KB resistance identified on 4B.

## Introduction

Wheat (*Triticum aestivum* L.) is consumed worldwide as a key component of the human diet. The global wheat production for 2018–19 was 730.9 million tons, which is approximately 29 million tons lower than production in the preceding year (http://www.fao.org/worldfoodsituation/csdb/en/). With the global population estimated to be 9 billion in 2050, wheat demand is expected to increase by 60%. Increases in the annual wheat yield must grow from the current level of less than 1% to at least 1.6% to meet the demand (https://www.wheatinitiative.org/about-us/wheat-research-food-security).

Rusts, smuts, bunts, mildews and foliar blights are major biotic factors that significantly affect wheat production and productivity. Karnal bunt (KB), which is caused by *Neovossia indica*, is one of five bunt and smut diseases of wheat and a serious concern to the grain industry due to quarantine regulations that restrict the international movement and trade of affected stocks. KB directly attacks the economic part, i.e., seed of the crop (Supplementary Fig. S1). After the attack, the fungus forms black teliospores in sori and deteriorates the grain quality by generating an unpleasant, and foul smell attributed to trimethylamine produced by the fungus^1^. The infection of approximately 4% of kernels is sufficient for the grain to become unfit for human consumption due to a low palatability^2^. Internationally, approximately 70 countries have placed an embargo on the import of Karnal bunt-infected wheat^3^. The European and Mediterranean Plant Protection Organization (EPPO) has categorized *N. indica* as a quarantine pest (zero tolerance), which forced the adoption of strict laws under the Sanitary and Phytosanitary (SPS) agreement^4^.

The management of KB is challenging due to long-lived teliospores^5^. As a seed-, soil- and air-borne fungus, the control of the disease through cultural practices and/or seed treatment is not very effective^6^. The chemical control of KB by spraying fungicides at the anthesis stage is possible but not cost-effective for farmers; thus, conventional methods are not sufficient to cope with KB^2,7^.

The identification and development of resistant genotypes is a viable option to manage KB^8^. However, the lack of fast, simple and environment-independent methods for screening wheat germplasm that resist the pathogen is a bottleneck during the development of resistant varieties. Researchers are managing the problem with available screening methods, but because KB is a disease of the mature grain, the identification of resistant donors is only possible in the next generation, which also decelerates the breeding progress. Because of these limitations, marker-aided selection/screening (MAS) is another opportunity for efficient introgression of resistance into elite genotypes^8^.

Linkage analyses and genome-wide association studies (GWAS) are two powerful statistical genetic methods that have been successfully deployed for the genetic dissection of quantitative traits^9^. The former relies on trait segregation in a bi-parental cross population and has been exploited for the identification of QTLs for KB resistance in a range of studies^3,8^. GWAS is a population-based method that exploits the linkage disequilibrium phenomenon between a trait and a genetic marker^10^. This mapping population-independent method relies on a population consisting of a set of unrelated accessions and has been successfully used to identify numerous traits in many crops, such as *Arabidopsis thaliana*^11^, rice^12^, barley^13^ and wheat^14^. However, only two GWAS studies have been reported to date with the aim of determining marker-trait associations (MTAs) for KB resistance in elite wheat lines^15,16^. Both these studies were carried out with wheat germplasm collection. Here, we report the very first association analysis of KB in the diverse wheat pre-breeding lines derived from exotic x elite crosses and elucidate the genetic components of KB resistance.

## Results and Discussion

KB is a quarantine disease (zero percent tolerance) that is indirectly responsible for economic losses in more than 40 countries by affecting the free import-export of wheat grains. Additionally, infected grains are not fit for human and animal consumption^17^. Thus, the identification and/or development of KB resistance genotypes(s) that minimize the probability of establishment or decrease the inoculum spread rate into newer areas are urgently needed^17^. The development of resistant cultivars through breeding programs is an appropriate strategy to achieve zero tolerance to the disease.

### Evaluation of pre-breeding germplasm for KB resistance

**Figure 1:**
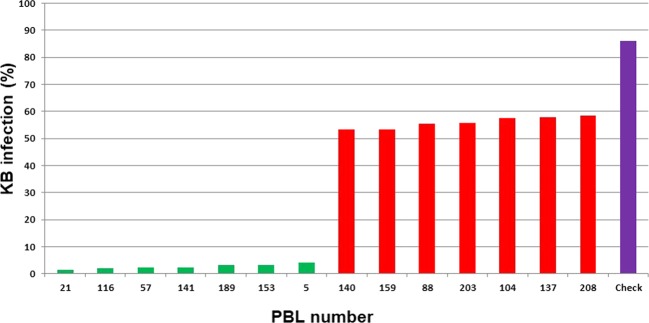
The severity of KB disease in seven highly resistant genotypes (green bar; 1.43–3.99% infection), seven highly susceptible genotypes (red bar; 53.38–58.34% infection) and KB-SUS check (purple bar; 86% infection). X-axis = name of the genotypes and check and Y-axis = KB infection (%).

**Figure 2:**
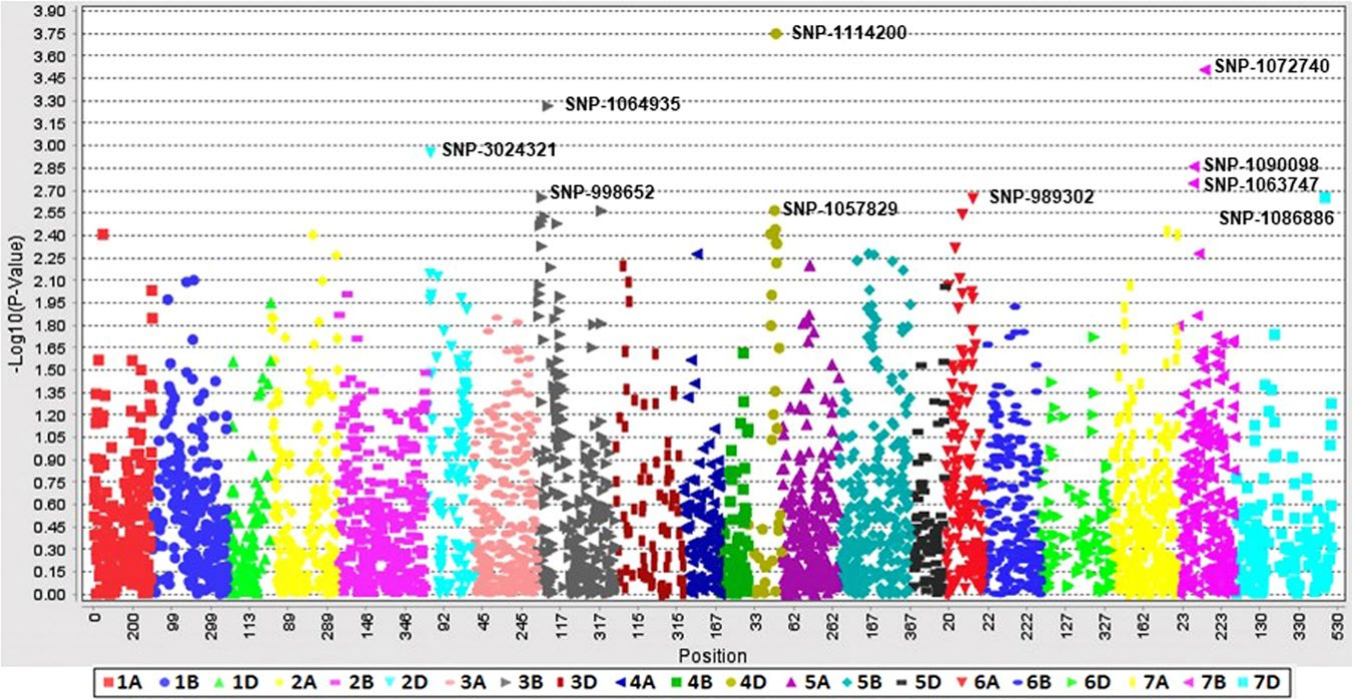
Genome-wide scan (GWAS analysis) of SNP markers associated with Karnal bunt resistance in 179 PBLs in environment 2 (2017–18). The plots show SNP-based Manhattan plots with the names of the most significant SNPs. The chromosomes are shown on the X-axis and the genome-wide scan −log^10^ (*P*-values) are shown on the Y-axis.

The sporadic incidence of KB in natural hotspots and the role of the environment in disease development make studies of KB resistance more challenging and time-consuming. Screening of wheat germplasm against the pathogen has been attempted by various researchers. Until 1979, screening experiments were conducted at KB hotspot locations. Subsequently, researchers developed an effective screening system, and since then, a large number of wheat germplasm and allied species have been screened under artificial epiphytotic conditions^18^. The severity of KB incidence in the germplasm of 179 pre-breedling lines from different environments ranged from 1.43–58.34%, whereas infection with KB-SUS (check) ranged from 53.38 to 98.84% (mean = 86.08 ± 12.49; Fig. 1). The mean severity was 26.09 ± 13.76%, indicating a significant difference in KB resistance among the studied genotypes at the genetic level. A summary of the results from the detailed phenotypic analysis is presented in Table 1 and Supplementary Table S1. The frequency distribution of the disease in the population lines is presented in Fig. 2 and indicated a trend towards a normal distribution. The severity of infection ranged from 0 to 69.03% in E-1 and from 0–56.92% in E-2. The mean value for infection with the disease in E-1 (28.26%) was greater than in E-2 (23.91%). The variation in disease resistance in the currently analyzed germplasm suggested that KB resistance was reinforced in different genetic backgrounds of the most appropriate high yielding varieties/genotypes. Therefore, PBLs with KB resistance can be used to create immunity for the disease^19^. This magnitude of genetic variation in infection indicated that a GWAS would likely reveal the underlying QTLs. During KB screening, breeders select genotypes that exhibit an infection rate less than 5% and exploit them in wheat improvement program. In current study with more stringency of selection, seven PBLs exhibited an infection severity of less than 4.0% at both E-1 and E-2 (Table 2 and Fig. 1). Variable levels of disease incidence for KB have been reported in different germplasm specimens by various researchers; for example, 1 to 48% and 2 to 95% disease infection rates were recorded in spring and durum wheat, respectively^20^. A previous study reported a percentage of disease incidence ranging from 0.2–63.1% among 150 wheat genotypes^21^. A significant correlation (0.81**) between KB infection and the year indicated that the disease pressure was substantial in both years and phenotypic data were suitable for the GWAS analysis (Supplementary Table S1).

**Table 1:** Mean, range and variance of the population evaluated for Karnal bunt disease infection in wheat in E-1 (2016–17), E-2 (2017–18) and joint analyses.

Environment	Mean±S.D.	Range	S.E.	Variance
E-1	28.26 ± 16.09	0.0–69.03	1.20	259.03
E-2	23.91 ± 12.78	0.0–56.92	0.96	163.37
Joint analysis	26.09 ± 13.76	0.0–58.34	1.03	188.20

**Table 2:** Details of the pedigree, Karnal bunt resistance level and the favorable allele of seven PBLs.

Entry	GID	Pedigree	KB infection (%) E-1 (2016–17)	KB infection (%) E-2 (2017–18)	KB infection (%) Joint analysis	Rank	Status of the favorable allele from SNP 1114200 (chr 4D)
21	7642410	IG 122628/SUP152//Villa Juarez F2009	2.43	0.43	1.43	1	No data
116	7641460	ARLIN_1/*Ae. squarrosa* (1017)//Kachu/3/Baj#1	2.87	1.21	2.04	2	T
57	7642476	BCN//CETA/*Ae. searsii* (34D)/3/Villa Juarez F2009/4/WBLL1/ Kukuna//Tacupeto F2001/3/Baj #1	2.33	2.23	2.28	3	T
141	7645911	68.111/RGB-U//WARD/3/FGO/4/RABI/5/ *Ae. squarrosa* (675)/6/NAVJ07/7/KACHU	3.14	1.54	2.34	4	T
189	7645914	68.111/RGB-U//WARD/3/FGO/4/RABI/5/*Ae. squarrosa* (675)/6/NAVJ07/7/KACHU	1.12	5.41	3.265	5	T
153	7645234	LOCAL RED/*Ae. squarrosa* (222)/3/Attila*2/PBW65//Murga/4/REEDLING #1	4.04	2.19	3.115	6	T
5	7642480	BCN//Ceta/*Ae.searsii* (34D)/3/Villa Juarez F2009/4/Wblli/Kukuna//Tacupeto F2001/3/Baj #1	6.53	1.46	3.995	7	T

The analysis of variance suggested that both the genotype and year exerted a significant effect on KB (Supplementary Table S2). Similar results were recorded during QTL mapping for KB resistance in two different studies^2,8^. The heritability of trait was high in both years (91% and 87% in E-1 and E-2, respectively) (Supplementary Table S3), suggesting that KB resistance can be mapped through QTL mapping and GWAS^2,15^. The phenotyping protocols established at CENEB, Cd. Obregon and reported in a previous study^22^ that limit environmental or unexplained variations account for the high heritability observed in the present and previous studies. The disease is highly influenced by environmental conditions, but in current investigation the environmental effect was not significant and high heritability of the disease was observed. The high level of genetic variation and high heritability recorded in the current study will be very useful for selection to genetically improve KB resistance in wheat, and highly resistant genotypes may be shared with partners to exploit them in KB-resistant breeding programs.

### Distribution frequency of KB disease in PBLs

The distribution frequency of disease infection was continuous in both environments, suggesting that KB resistance in wheat displays a quantitative inheritance pattern (Supplementary Fig. S2). The continuum of resistance against KB in the germplasm of wheat is well-established in previous studies^8,23^ and our study corroborates these findings. Moreover, the distribution of KB infections was skewed towards low disease severity, indicating that some major effect-related genes or genes involved in epistatic effects controlled KB resistance in the population investigated.

### Distribution of SNP markers, Genetic Diversity and Population Structure

A total 6,382 of high-quality SNPs were obtained from the 179 PBLs and were subjected to genetic diversity, population structure and GWAS analyses. The analysis of the genotype data revealed differences in the distribution of polymorphic DArT markers among the A, B, and D genomes (Supplementary Fig. S3). The B genome contained the greatest number of SNPs (2349, 36.80%), followed by the D (2038, 31.93%) and A genomes (1995, 31.25%). The D genome in wheat contains comparatively fewer polymorphic markers than the A and B genomes due to low recombination attributed to its evolutionary and domestication history. Consistent with previous findings, the B genome contains a greater number of polymorphisms than the A and D genomes, which is attributed to a greater number of effective recombination events in the B genome^24–26^. The average number of SNPs per chromosome was 304, and the value ranged from 147 (4B) to 500 (7D) (Supplementary Fig. S4).

The ratio of the number of SNPs in the B and A genomes was 1.17, 1.15 in the B and D genomes, and 0.98 in the A and D genomes. Based on these ratios, the number of SNPs in the D genome was approximately equal to the number of SNPs in the A genome and only slightly less than the number of SNPs in the B genome. A similar ratio was also recorded between the A and D genomes^27^. Many researchers have reported a high value for the ratios between D and A or B genomes, but this finding was not corroborated in the current study, indicating the high variability of D genome in PBLs. As described in a previous study, unique genetic variations and wild relative genes can be used to broaden the genetic base of wheat, extend the genetic gain and weaken the genetic bottlenecks associated with the D genome^28^.

The mean genetic diversity in current sets of germplasm was 0.32 (range 0.035–0.71), which is higher than previous studies examining SNP markers^29,30^. The high value of genetic diversity may be attributed to the exploitation of diverse exotic parental lines to generate PBLs. The mean genetic diversity value was close to the value reported for the Wheat Association Mapping Initiative (WAMI) population (0.31), which comprises many synthetically derived lines. The PIC (0.28; range 0.01–0.59) of the current study was comparatively higher than values (0.21, 0.16) reported in previous studies^31,32^.

The population structure can distort the MTA by revealing false positive associations in a GWAS. Therefore, information on the population structure is a prerequisite^33^. The largest delta K was observed at K = 6, suggesting the presence of six sub-populations (Supplementary Fig. S5). The maximum number of genotypes were grouped in sub-population 3 (83 PBLs) followed by sub-population 5 (44 PBLs). Sub-population 4 included two PBLs, while sub-populations 1, 2 and 6 contained 10, 13 and 27 PBLs, respectively.

Two elite lines (Baj #1 and Super 152) in the parentage dominated sub-population 1, as 80% of the population lines had these parents. Seventy percent of the lines in subpopulation 2 shared three elite parents, Wiblli, Kukuna and Tacupeto F2001, while the remaining lines shared two parents, Baj #1 and Super152. Similarly, sub-populations 3, 4, 5 and 6 were dominated by parents Baj#1, Super152/Villa Juarez F2009, Kachu and Kiritati, respectively. Based on these results, sub-populations largely corresponded to the elite parents in each cross of exotic/elite1/elite2.

### Linkage disequilibrium analysis and genome-wide association study of KB resistance

The extent of LD decay in this panel at cut off *r*^2^ = 0.1 has been reported to 2.5 cM for whole genome^34^, which suggests higher genetic diversity of the investigated panel as compared to the wheat panels used in previous studies^31,35–37^. This panel has been drawn from a large set of pre-breeding lines (PBLs) developed by a three way crossing scheme (exotic/elite1//elite2) among exotics and elites^38^. Each pre-breeding line acquired approximately 25% of the exotic genome and 75% of the elite genome at an early stage thus allowing recombination between exotic and elite genomes. Further, LD decay in the three genomes was at 2.5, 5.0, and 2.5 cM for A, B, and D genomes, respectively. These results indicate a faster LD decay in the D genome, almost comparable to A genome, than reported in previous studies. This might be due to the use of synthetics for developing PBLs, driving more recombination in the D genome.

**Figure 3:**
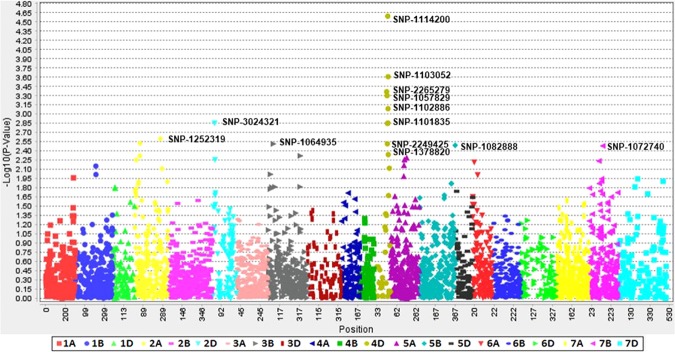
Genome-wide scan (GWAS analysis) of SNP markers associated with Karnal bunt resistance in 179 PBLs in environment 1 (2016–17). Plots show SNP-based Manhattan plot with the names of the most significant SNPs. The chromosomes are shown on the X-axis and the genome-wide scan −log^10^ (*P*-values) are shown on the Y-axis.

With MLM (PCA + K), 15 significant SNPs on chromosomes 2D (1 SNPs), 3B (4 SNPs), 4D (7 SNPs), 5B (1 SNP), 6A (1 SNP) and 7B (1 SNP) were identified with *P*-values <10^−3^ in the joint analysis (Supplementary Fig. S6) and explained 5.48 to 12.49% of the phenotypic variation (Table 3, Supplementary Table S4). Significant marker-trait associations at the 0.05 FDR threshold in E1, E2, and joint analysis are presented in Table 3. Consistent and common associations were observed on chromosomes 2D, 3B, 4D and 7B based on FDR values. In the current study, the year exerted a significant effect on disease expression and the same trend was also recorded in the analysis of marker-trait associations, as many of SNPs were not shared between the years. In individual years, 13 significant SNPs were detected in E-1 (Fig. 3), while 10 significant SNPs were detected in E-2 (Fig. 2). Among  E-1, E-2 and the joint analysis, five SNPs (2D/0.139 cM, 3B/0.562 cM, 4D/1.496 cM, 4D/1.571 cM, and 7B/0.952 cM) were confirmed to be common and explained between 7.5 to 12.49% of the variation  (Table 3).

**Table 3:** Marker trait associations for resistance to karnal bunt in wheat.

Environment*	Marker	Chr	Pos	*P*-value**	FDR adjusted *P* values*** (p < 0.05)	R^2^
Joint analysis	1114200|F|0--63:T>C	4D	1.572	7.97E-06	3.00E-04	0.12
Joint analysis	1103052|F|0--61:C>A	4D	1.574	1.98E-04	0.002	0.09
Joint analysis	2249425|F|0--21:T>C	4D	1.540	2.10E-04	0.002	0.07
Joint analysis	3024321|F|0--17:T>C	2D	0.392	2.25E-04	0.001	0.07
Joint analysis	1072740|F|0--54:G>C	7B	0.952	2.54E-04	0.001	0.08
Joint analysis	2265279|F|0--16:T>C	4D	1.473	2.82E-04	0.001	0.07
Joint analysis	1057829|F|0--25:G>A	4D	1.496	3.07E-04	0.001	0.07
Joint analysis	1102886|F|0--29:A>G	4D	1.574	3.18E-04	0.001	0.08
Joint analysis	1064935|F|0--7:G>C	3B	0.562	4.50E-04	0.001	0.07
Joint analysis	1101835|F|0--5:C>A	4D	1.574	4.72E-04	0.001	0.07
Joint analysis	1006701|F|0--18:C>T	6A	0.332	9.34E-04	0.002	0.06
Joint analysis	998652|F|0--18:T>C	3B	0.334	0.00169	0.003	0.05
Joint analysis	999156|F|0--6:T>C	5B	2.365	0.00196	0.003	0.05
Joint analysis	1241181|F|0--46:T>C	3B	0.372	0.00202	0.003	0.05
Joint analysis	1095156|F|0--52:C>T	3B	2.344	0.00205	0.003	0.06
E-1	1114200|F|0--63:T>C	4D	1.572	2.54E-05	4.82E-04	0.11
E-1	1103052|F|0--61:C>A	4D	1.574	2.46E-04	0.001	0.09
E-1	2265279|F|0--16:T>C	4D	1.473	4.33E-04	0.001	0.08
E-1	1057829|F|0--25:G>A	4D	1.496	5.02E-04	0.001	0.08
E-1	1102886|F|0--29:A>G	4D	1.574	8.19E-04	0.002	0.08
E-1	3024321|F|0--17:T>C	2D	0.392	0.0014	0.002	0.06
E-1	1101835|F|0--5:C>A	4D	1.574	0.0014	0.002	0.06
E-1	2249425|F|0--21:T>C	4D	1.540	0.00142	0.002	0.06
E-1	1252319|F|0--6:T>C	2A	2.174	0.00252	0.003	0.06
E-1	1378820|F|0--21:G>A	4D	1.540	0.00305	0.003	0.06
E-1	1064935|F|0--7:G>C	3B	0.562	0.00306	0.003	0.06
E-1	1082888|F|0--6:T>G	5B	2.942	0.00323	0.003	0.06
E-1	1072740|F|0--54:G>C	7B	0.952	0.0033	0.003	0.06
E-2	1114200|F|0--63:T>C	4D	1.572	1.78E-04	0.002	0.09
E-2	1072740|F|0--54:G>C	7B	0.952	3.12E-04	0.001	0.08
E-2	1064935|F|0--7:G>C	3B	0.562	5.43E-04	0.001	0.07
E-2	3024321|F|0--17:T>C	2D	0.392	0.00111	0.002	0.06
E-2	1090098|F|0--31:A>G	7B	0.823	0.00138	0.002	0.06
E-2	1063747|F|0--8:C>T	7B	0.823	0.00179	0.003	0.06
E-2	998652|F|0--18:T>C	3B	0.334	0.00222	0.003	0.05
E-2	1086776|F|0--6:T>G	7D	3.464	0.00223	0.003	0.11
E-2	989302|F|0--48:A>C	6A	1.229	0.00225	0.003	0.05
E-2	1057829|F|0--25:G>A	4D	1.496	0.00272	0.003	0.05

*E-1 = Environment 2016–17, and E-2 = Environment 2017–18, **P values obtained from model MLM, ***FDR adjusted *P* values at P < 0.05.

Previous studies reported the presence of KB resistance loci scattered on many chromosomes in all three genomes of wheat^15,16^. Hence, cultivar pyramiding/stacking of favourable alleles from multiple loci is a prerequisite to develop KB resistance^22^.

**Figure 4:**
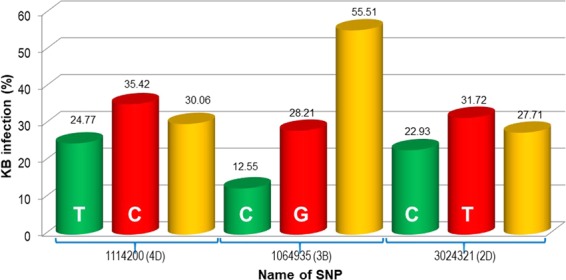
Effect of alleles of significant SNPs (common in three datasets) on KB severity (green bar = favorable allele providing resistance; red bar = unfavorable allele; yellow bar = % increase in resistance if favorable allele introgressed). X-axis = name of SNPs and Y-axis = infection level (%).

The results of the current study validated the consensus region on chromosome 3B reported in previous studies^2,15^ (Fig. 4). Likewise, QTLs for KB resistance on chromosomes 5B and 2D^8,15^ were also reported previously in RIL populations and a GWAS panel, respectively. A marker-trait association was also detected on chromosome 7B, which might be the same locus that was previously reported^23^. Interestingly, seven SNPs were detected on chromosome 4D. Two of these SNPs had maximum *R*^*2*^ values: SNP (1114200 | F | 0–63:T > C) at position 1.571 cM with an *R*^*2*^ of 12.49% and SNP (1103052 | F | 0–61:C > A) at position 1.574 cM with an *R*^*2*^ of 9.02%. The physical positions of SNPs, sequence reads of the SNPs were blasted to the reference genome of RefSeq V.1.0 with E value <10^−8^ and identity >95%^38^. These two SNPs were in strong LD with each other, confirming that they are part of the same genomic region on chromosome 4D (Supplementary Fig. S7). No previous studies have reported loci for KB resistance on chromosome 4D, suggesting that the D genome should be targeted for an in-depth investigation to map new KB resistant loci.

The donor of the D genome, i.e., *Aegilops tauschii*, in hexaploid wheat has been a source of useful genes, for example, rust resistance genes^23^. In the current study, two KB resistant loci were located on the D genome (chromosomes 2D and 4D) (Fig. 4). The wider genetic variation derived from synthetics and the diverse genetic make-up of PBLs obtained from populations used in previous studies may be the probable explanation for the identification of novel KB resistance loci on the D genome.

Chromosome 3B appears to be a hot spot for various disease resistance genes, as it is the location of QTLs for KB resistance^24^ and resistance to tan spot^39^, yellow rust^39^, leaf rust^24^ and crown rot^26^. Genomic regions inherited as multi-disease resistance loci are not uncommon in wheat^39^. Therefore, researchers should focus on chromosome 3B for future introgression breeding to create cultivars with simultaneous resistance to multiple diseases. Likewise, the current GWAS panel may be screened for pathogens causing devastating diseases, such as a blast to identify major effect QTLs and/or to reveal genomic hot spot(s) for multi-disease resistance.

During the detection of highly tolerant PBLs based on phenotypic data, a set of 7 lines were confirmed to be resistant (Table 2). An analysis of the pedigree of these 6 PBLs revealed that *Aegilops* species are one parent used during the creation of synthetic wheat and contributed the D genome to these resistant PBLs. The analysis of these same lines for the SNP 1114200 marker (chromosome 4D) indicated that 6 of 7 PBLs contained the ‘T’ allele (favorable allele contributing to resistance). The role of the D genome in KB resistance suggested that the D genome must be further enriched with additional polymorphic SNPs to exploit more QTLs for various diseases viewed as upcoming threats to wheat cultivation.

During the study, SNPs on chromosome 4D were not associated with the *Rht-D1* dwarfing locus, as the frequency of the dwarf allele was 0.1% (2 of 179 PBLs exhibited wild allele, i.e., G), indicating that the *Rht-D1* gene was fixed  in the current KB panel of PBLs and the KB resistance locus on chromosome 4D is novel (Supplementary Table S5).

### Significance of chromosome 4D

Seven SNP markers on chromosome 4D were associated with KB infection in the joint analysis (Table 3). Noticeably, all but one SNP were present in strong LD (Supplementary Fig. S7). We divided the panel into two groups. Group 1 (carrying all favourable alleles of 7 markers) contained 109 PBLs and group 2 (carrying unfavourable alleles for any of the 7 markers) contained 70 PBLs. The mean KB% was 12.16% higher than in group 1. This comparison further confirms that the allelic profile of group 1 PBLs is associated with resistance to KB (Supplementary Fig. S8). However, an in-depth analysis of a set of diverse exotic and elite accessions should improve our understanding of this genomic region on chromosome 4D.

The identified markers were subjected to BLAST search in Ensembl Plants server (https://plants.ensembl.org/Triticum_aestivum/Info/Index) to obtain additional insights into the genes on chromosome 4D that are involved in KB resistance. Of the 8 associated SNPs located on chromosome 4D, three showed direct hits with candidate genes and three were located in the vicinity (2 Mb) (Supplementary Table S6). Of the three direct hits, two hits with markers 1103052 | F | 0–61:C > A (TraesCS4D02G352200) and 1101835 | F | 0–5:C > A (TraesCS4D02G350300) were interesting, belonging to RBOHB and Rhomboid-like protein gene families^40,41^. The gene TraesCS4D02G352200 showed the closest relationship to the AET4Gv20828800 gene in *Aegilops tauschii* (Supplementary Fig. S9). One of the molecular functions of TraesCS4D02G352200 is NAD(P)H oxidase activity. As key producers of reactive oxygen species, NADPH oxidases (NOXs), which are also known as respiratory burst oxidase homologs (RBOHs), play crucial roles in various biological processes in plants. Two TaNOX genes have been reported to confer resistance to brown rust infection^40^. To date, 15 TaNOXs have been reported to be co-expressed with different sets of other genes that participate in several critical intracellular processes such as cell wall biosynthesis, defence response, and signal transduction, suggesting vital but diverse roles for these genes in regulating plant growth and stress responses in wheat^43^. Two of these genes (*TaNOX8* and *TaNOX9*) were mapped to chromosome 4DL. TaNOX8 is specifically present on the membrane and is co-expressed with *TaNOX13AL* on chromosome 5AL. Thus, we speculate that the marker 1103052 | F | 0–61:C > A might represent the *TaNOX8* gene. The other candidate gene, TraesCS4D02G350300, is a Rhomboid-like protein, which belongs to a family of serine proteases (family S54) that cleave substrates within transmembrane domains^44^. Rhomboid protease family member S54 has proven to be active during fungal-plant interactions^45^.

**Figure 5:**
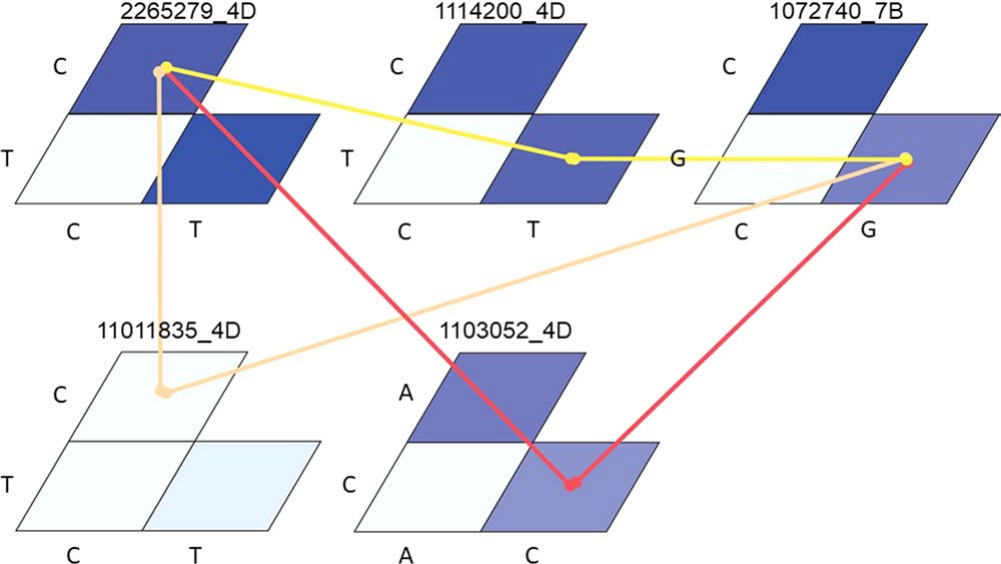
Epistatic interactions among loci associated with KB resistance in wheat. The magnitude of the marker effect (F value) is represented in shades of blue (dark blue indicates a stronger interaction). The magnitude of the epistatic interaction is presented with colors ranging from yellow (stronger interaction) to red (weaker interaction).

Furthermore, these two markers, 1103052 | F | 0–61:C > A and 1101835 | F | 0–5:C > A, on chromosome 4D were significantly involved in epistatic interactions with other loci on chromosome 4D and with the associated locus on chromosome 7B (1072740 | F | 0–54:G > C) (Fig. 5), contributing an additional 8% to the variation. Therefore, KB resistance is controlled by minor additive and epistatic loci. Previous studies using bi-parental populations and a GWAS panel have reported additive QTLs for KB resistance^2,15,16^, but information on epistatic QTL is lacking. This report is the first to identify additive and epistatic QTL for KB resistance in a GWAS panel. A similar level of interaction was also reported for APR loci for stem rust in wheat^46^.

## Materials and methods

### Plant material

The plant material used in the current investigation comprised 179 pre-breeding lines (PBLs). A detailed description of the development of these PBLs has been reported in Singh *et al*.^38^. Briefly, a set of 179 outstanding PBLs were selected from a larger set of 984 TC_1_F_5:6_ based on their tolerance to biotic and abiotic stresses, namely, heat, drought, yellow rust (*Puccinia striiformis* f. sp. *tritici*), and powdery mildew (*Blumeria graminis* f. sp*. tritici* (Bgt)), character and yield performance in Mexico. The pedigree information for these lines is presented in Supplementary Table S7. KB-SUS was used as a susceptible line and grown around all of the experimental blocks.

### Phenotyping PBLs for Karnal bunt disease through artificial inoculation methods

The phenotypic screening was conducted at Ciudad Obregon, Mexico over two years (2016–17 was referred to as E-1 and 2017–18 was referred to as E-2) using a randomized complete block design (RCBD). For the preparation of the inoculation, one-year-old teliospores were scraped off infected grains with a dissecting needle and incubated in a water-Tween 20 solution for 24 h; then, the suspension was filtered through a 60 µM nylon sieve and centrifuged at 3000 rpm. After discarding the supernatant, 0.5% sodium hypochlorite was applied as the active ingredient for 2 min to disinfect teliospores and the suspension was centrifuged again. Teliospores were then rinsed twice with sterile distilled water, followed by centrifugation. Teliospores were resuspended in sterile distilled water in the centrifuge tube. One milliliter of the teliospore suspension was spread on Petri dishes containing 2% water-agar (AA) and incubated at 18–22 °C in the dark. After 6 to 9 days, teliospore germination was evaluated using a compound microscope at 10x magnification. Pieces of AA with germinated teliospores were removed and placed upside down on the lid of Petri dishes containing potato-dextrose-agar (PDA). After 10 to 14 days, 2 to 3 mL of sterile distilled water was added to the plates and the colonies were scraped gently using a sterile spatula. Hyphae and sporidia were inoculated onto other plates with PDA using a sterile syringe, and the plates were incubated at 18–22 °C in the dark for approximately nine days. After the incubation, pieces of PDA with the different fungal propagules were transferred and placed upside down on the lids of sterile glass Petri dishes to induce the production of allantoid secondary sporidia^47,48^. Three milliliters of sterile distilled water were added to the bottom of the plates.

Water was collected from the plates every 24 h; secondary allantoid sporidia were collected and counted using a hemocytometer. Then, the concentration was adjusted to 10,000 sporidia per mL. Five heads of each experimental line at the boot stage were inoculated by injecting 1 mL of the allantoid sporidial suspension during the boot stage. Stems of the inoculated heads were identified with a piece of red plastic. An automatic mist irrigation system was used to water the plants during the period of inoculation (January-March) for 20 min five times per day, and the area was covered with nets to prevent bird damage. Grains were harvested manually, and the healthy and infected grains were counted by a visual inspection to calculate the percentage of infection (infected grains). Standard agronomic practices were used during the crop season. The mean disease incidence (%) was estimated from five inoculated spikes per plot. The phenotyping for KB screening was based on ratings reported as percentages to identify tolerant and susceptible genotypes^2^.

The mean for each PBL was calculated to reduce the environmental variance in the data recorded through the partitioning of phenotypic variance using the maximum residual likelihood (ReML) algorithm. The KB infection data were subjected to an ANOVA to reveal the effects of the genotype, year and their interaction.

### High-throughput genotyping

A modified CTAB (cetyltrimethylammonium bromide) method was used to extract genomic DNA from leaf samples^49^. Leaves were collected from each PBL (TC_1_F_5_) 25 days after germination. The extracted DNA was quantified with a NanoDrop 8000 spectrophotometer V 2.1.0, while intactness was analyzed using agarose gel electrophoresis. The DNA concentration was adjusted to 50–100 ng µL^−1^ in a volume of 30 µL. Genotypic characterization used DArTseq technology (http://www.diversityarrays.com/dart-application-dartseq) at the Genetic Analysis Service for Agriculture(SAGA) service unit at CIMMYT headquarters (Texcoco, Mexico). The main parameters to select markers were call rate (the proportion of samples with genotypic score and not recorded as missing data) and average reproducibility (the proportion of technical replicate assay pairs for which the marker score was consistent). More details on SNP filtering are given in Ledesma-Ramírez^34^. A total of 12,071 SNP markers belonging to 10,111 sequence tags were identified based on above criteria. Chromosome location, marker order and genetic distances were defined based on a 64,000-marker DArT-seq consensus map released by Diversity Arrays Technology Pty Ltd. (DArT) (http://www.diversityarrays.com/sequence-maps). The methodology described by Vikram *et al*.^49^ was followed to capture 6,382 high quality SNP markers on 179 PBLs. These high-quality DArTseq SNPs were used for the population structure and association analyses (Supplementary Table S8).

### Population Structure and Linkage Disequilibrium (LD) Analysis

The structure was analyzed using K-values ranging from 1 to 8 for the entire population with 6,382 SNPs markers with *STRUCTURE* software^51^. Five independent analyses were performed for each K-value, with 100,000 set as the length of the burn-in time and number of Markov Chain Monte Carlo replications. The correct estimation of K was provided by an *ad hoc* statistic deltaK^52^ that was calculated using the program STRUCTURE HARVESTER^53^. The linkage disequilibrium analysis was conducted on this panel using GAPIT version 2.0^34^. Birefly, a squared correlation coefficient *r*^2^ was estimated for all pairwise comparisons. Pattern of LD decay was then visualized by plotting pair-wise *r*^2^ values against the genetic distance for A, B, and D genomes separately and for whole genome. For the LOESS estimation of LD decay, genetic distance was estimated as the point where the LOESS curve first crosses the baseline *r*^2^ of 0.1.

### Association analysis

Phenotypic data from two years were analyzed separately and combined for the association analysis. TASSEL software v5.01^54^ was used to calculate associations between the markers and KB score with the mixed linear model (MLM)^55^. MLM takes advantage of the population structure (Q) and kinship (K) matrix as covariates during a GWAS to avoid spurious associations^56^. Both of these matrices were also generated using TASSEL 5.01. To correct for multiple testing, the step up procedure of Benjamini and Hochberg^29^ which controls the false discovery rate was used with a cut-off value of 0.05. ANOVA analysisand estimates ofcorrelations and heritability were conducted in SAS 9.4. The KB scoing data of both environments separately and mean over the environments (Supplementary Table S9**)** was used for the association mapping.

### Epistatic interactions

Two- and three-locus interactions among associated loci were studied using an in-house script executed in R, as described previously^31,42,50^. A significant threshold of *P* < 0.001 represented significant marker-marker interactions.

### *In silico* analysis

*In silico* analysis of the significant loci was conducted in *Ensembl Plants* sequence database (https://plants.ensembl.org/Triticum_aestivum/Info/Index) by using BLASTN algorithm with functional annotations from IWGSC v1.1 RefSeq annotation. Precisely, to find the candidate genes, the physical starting point of the marker along with chromosome name was put in *Ensembl Plants* and around 300 bp were added before and after the SNP. For some markers, SNPs fell within gene sequences and hence were classified as direct gene hits in Supplementary Table S6. However, for markers where SNPs did not fall within a gene, potential candidate genes were picked 2 Mb upstream and downstream of the SNPs which were related to pathogenic process or known to regulate induction of genes related to pathogenesis. Sometimes annotations were not available in *Triticum aestivum* genome and for such cases orthologous genes in related species were screened with known predicted functions using the comparative genomics tool in Ensembl.

## Conclusions

In the present study, an association mapping panel consisting of 179 PBLs genotyped with 6382 high-quality DArTseq SNP markers was utilized to obtain an understanding of the genetics of Karnal bunt resistance in wheat (Supplementary Fig. S10). Fifteen significant SNPs were identified on chromosomes 2D, 3B, 4D and 7B in a joint analysis of data collected from 2 years. This report is the first to describe QTLs/genes associated with KB resistance on chromosome 4D. The SNPs associated with KB resistance might be exploited through marker-assisted selection to facilitate the breeding of resistant wheat. Eventually, PBLs carrying more favourable alleles coupled with superior agronomic performance could be utilized as excellent parental materials to produce improved wheat lines that resist this stress. These results provide new information for further research aiming to improve KB resistance in common wheat. Moreover, the 7 best identified resistant lines have become part of the breeding programs of many Asian countries (India and Pakistan) where KB is a major problem.

## Supplementary information

Supplementary material.

Supplementary material 2.

Supplementary material 3.

Supplementary material 4.

Supplementary material 5.

Supplementary material 6.

Supplementary material 7.

Supplementary material 8.

Supplementary material 9.

Supplementary material 10.

Supplementary material 11.

Supplementary material 12.

Supplementary material 13.

Supplementary material 14.

Supplementary material 15.

Supplementary material 16.

Supplementary material 17.

Supplementary material 18.

Supplementary material 19.

figure legends
