# Supplementary material for: GWAS revealed a novel resistance locus on chromosome 4D for the quarantine disease Karnal bunt in diverse wheat pre-breeding germplasm
Source: Sci Rep. 2020 Apr 7;10:5999. doi: 10.1038/s41598-020-62711-7 (PMC7138846; doi:10.1038/s41598-020-62711-7)
Supplement: Supplementary file 1 — Supplementary material. [file 41598_2020_62711_MOESM1_ESM.docx]

**Supplementary table S4** Marker trait associations for Karnal Bunt in wheat

| **Environment*** | **Marker** | **Chr** | **Pos** | **F** | ***P*-value**** | **R^2^** |
| --- | --- | --- | --- | --- | --- | --- |
| Joint analysis | 1114200\|F\|0--63:T>C | 4D | 1.571724286 | 21.39 | 7.97E-06 | 0.12 |
| Joint analysis | 1103052\|F\|0--61:C>A | 4D | 1.57403 | 14.55 | 1.98E-04 | 0.09 |
| Joint analysis | 2249425\|F\|0--21:T>C | 4D | 1.53954 | 14.35 | 2.10E-04 | 0.07 |
| **Joint analysis** | **3024321\|F\|0--17:T>C** | **2D** | **0.391743432** | **14.27** | **2.25E-04** | **0.07** |
| **Joint analysis** | **1072740\|F\|0--54:G>C** | **7B** | **0.95219** | **14.02** | **2.54E-04** | **0.08** |
| **Joint analysis** | **2265279\|F\|0--16:T>C** | **4D** | **1.47307** | **13.81** | **2.82E-04** | **0.07** |
| Joint analysis | 1057829\|F\|0--25:G>A | 4D | 1.49606 | 13.62 | 3.07E-04 | 0.07 |
| Joint analysis | 1102886\|F\|0--29:A>G | 4D | 1.57403 | 13.57 | 3.18E-04 | 0.08 |
| **Joint analysis** | **1064935\|F\|0--7:G>C** | **3B** | **0.562224549** | **12.91** | **4.50E-04** | **0.07** |
| Joint analysis | 1101835\|F\|0--5:C>A | 4D | 1.57403 | 12.73 | 4.72E-04 | 0.07 |
| Joint analysis | 1006701\|F\|0--18:C>T | 6A | 0.3324 | 11.35 | 9.34E-04 | 0.06 |
| Joint analysis | 998652\|F\|0--18:T>C | 3B | 0.33406 | 10.20 | 0.00169 | 0.05 |
| Joint analysis | 999156\|F\|0--6:T>C | 5B | 2.36466 | 9.94 | 0.00196 | 0.05 |
| Joint analysis | 1241181\|F\|0--46:T>C | 3B | 0.37233 | 9.84 | 0.00202 | 0.05 |
| **Joint analysis** | **1095156\|F\|0--52:C>T** | **3B** | **2.34353** | **9.87** | **0.00205** | **0.06** |
| E-1 | 1114200\|F\|0--63:T>C | 4D | 1.571724286 | 18.88 | 2.54E-05 | 0.11 |
| E-1 | 1103052\|F\|0--61:C>A | 4D | 1.57403 | 14.10 | 2.46E-04 | 0.09 |
| **E-1** | **2265279\|F\|0--16:T>C** | **4D** | **1.47307** | **12.93** | **4.33E-04** | **0.08** |
| E-1 | 1057829\|F\|0--25:G>A | 4D | 1.49606 | 12.62 | 5.02E-04 | 0.08 |
| E-1 | 1102886\|F\|0--29:A>G | 4D | 1.57403 | 11.65 | 8.19E-04 | 0.08 |
| **E-1** | **3024321\|F\|0--17:T>C** | **2D** | **0.391743432** | **10.58** | **0.0014** | **0.06** |
| E-1 | 1101835\|F\|0--5:C>A | 4D | 1.57403 | 10.56 | 0.0014 | 0.06 |
| E-1 | 2249425\|F\|0--21:T>C | 4D | 1.53954 | 10.52 | 0.00142 | 0.06 |
| E-1 | 1252319\|F\|0--6:T>C | 2A | 2.174446154 | 9.46 | 0.00252 | 0.06 |
| E-1 | 1378820\|F\|0--21:G>A | 4D | 1.53954 | 9.06 | 0.00305 | 0.06 |
| **E-1** | **1064935\|F\|0--7:G>C** | **3B** | **0.562224549** | **9.08** | **0.00306** | **0.06** |
| E-1 | 1082888\|F\|0--6:T>G | 5B | 2.942225 | 8.96 | 0.00323 | 0.06 |
| **E-1** | **1072740\|F\|0--54:G>C** | **7B** | **0.95219** | **8.90** | **0.0033** | **0.06** |
| E-2 | 1114200\|F\|0--63:T>C | 4D | 1.571724286 | 14.77 | 1.78E-04 | 0.09 |
| **E-2** | **1072740\|F\|0--54:G>C** | **7B** | **0.95219** | **13.60** | **3.12E-04** | **0.08** |
| **E-2** | **1064935\|F\|0--7:G>C** | **3B** | **0.562224549** | **12.52** | **5.43E-04** | **0.07** |
| **E-2** | **3024321\|F\|0--17:T>C** | **2D** | **0.391743432** | **11.04** | **0.00111** | **0.06** |
| E-2 | 1090098\|F\|0--31:A>G | 7B | 0.82303 | 10.61 | 0.00138 | 0.06 |
| E-2 | 1063747\|F\|0--8:C>T | 7B | 0.82303 | 10.09 | 0.00179 | 0.06 |
| E-2 | 998652\|F\|0--18:T>C | 3B | 0.33406 | 9.67 | 0.00222 | 0.05 |
| E-2 | 1086776\|F\|0--6:T>G | 7D | 3.463962 | 10.08 | 0.00223 | 0.11 |
| E-2 | 989302\|F\|0--48:A>C | 6A | 1.22863 | 9.66 | 0.00225 | 0.05 |
| E-2 | 1057829\|F\|0--25:G>A | 4D | 1.49606 | 9.27 | 0.00272 | 0.05 |

*E-1 = Environment 2016-17, and E-2 = Environment 2017-18, ***P* < 0.001, SNP highlighted

as bold are also significant based on FDR *P*-values provided in table 3.
