# Supplementary material for: GWAS revealed a novel resistance locus on chromosome 4D for the quarantine disease Karnal bunt in diverse wheat pre-breeding germplasm
Source: Sci Rep. 2020 Apr 7;10:5999. doi: 10.1038/s41598-020-62711-7 (PMC7138846; doi:10.1038/s41598-020-62711-7)
Supplement: Supplementary file 2 — Supplementary material 2. [file 41598_2020_62711_MOESM2_ESM.docx]

Table S1: Correlation analysis among Karnal bunt disease infection scores in E-1 (2016-17), E-2 (2017-18) and joint analyses

| Environment | Correlation | E-1 | E-2 |
| --- | --- | --- | --- |
| E-2 | Pearson | 0.81** |  |
|  | Spearman (rank) | 0.81** |  |
| Joint analysis | Pearson | 0.96** | 0.94** |
|  | Spearman (rank) | 0.96** | 0.94** |

** Significant at a *P* value = 0.01
