# Supplementary material for: GWAS revealed a novel resistance locus on chromosome 4D for the quarantine disease Karnal bunt in diverse wheat pre-breeding germplasm
Source: Sci Rep. 2020 Apr 7;10:5999. doi: 10.1038/s41598-020-62711-7 (PMC7138846; doi:10.1038/s41598-020-62711-7)
Supplement: Supplementary file 3 — Supplementary material 3. [file 41598_2020_62711_MOESM3_ESM.docx]

Table S2: Mean squares for Karnal bunt in PBLs from wheat in E-1 (2016-17) and E-2 (2017-18) in Mexico

| Source of variation | DF^ | Joint | E-1 | E-2 |
| --- | --- | --- | --- | --- |
| Env | 1 | 3408.13** | - | - |
| Rep(Env) | 2 (1) | 787.66** | 1020.81** | 554.51** |
| Genotype | 177 (177) | 716.53** | 492.71** | 311.20** |
| Env × Genotype | 177 | 84.68** | - | - |

^Figures in parentheses indicate degrees of freedom for individual environments.

** Significant at a *P* value = 0.01
