# Supplementary material for: GWAS revealed a novel resistance locus on chromosome 4D for the quarantine disease Karnal bunt in diverse wheat pre-breeding germplasm
Source: Sci Rep. 2020 Apr 7;10:5999. doi: 10.1038/s41598-020-62711-7 (PMC7138846; doi:10.1038/s41598-020-62711-7)
Supplement: Supplementary file 4 — Supplementary material 4. [file 41598_2020_62711_MOESM4_ESM.docx]

Table S3: Genotype variance (σ2 g), error variance (σ2 e), G x E variance (σ2 g × E) and heritability (H^2^ %) of Karnal bunt disease in E-1 (2016-17), E-2 (2017-18) and joint analyses

| KB | E-1 | | | E-2 | | | Joint analysis | | | |
| --- | --- | --- | --- | --- | --- | --- | --- | --- | --- | --- |
|  | σ ^2^ g | σ ^2^ e | H^2^ (%) | σ ^2^ g | σ ^2^ e | H^2^ (%) | σ ^2^ g | σ ^2^ e | σ ^2^ g × E | H^2^ (%) |
|  | 223.43 | 58.10 | 88.49 | 133.89 | 46.72 | 85.14 | 157.88 | 53.80 | 14.33 | 88.45 |
