# Supplementary material for: GWAS revealed a novel resistance locus on chromosome 4D for the quarantine disease Karnal bunt in diverse wheat pre-breeding germplasm
Source: Sci Rep. 2020 Apr 7;10:5999. doi: 10.1038/s41598-020-62711-7 (PMC7138846; doi:10.1038/s41598-020-62711-7)
Supplement: Supplementary file 5 — Supplementary material 5. [file 41598_2020_62711_MOESM5_ESM.docx]

Table S6: BLAST results for the markers located on chromosome 4D associated with KB resistance

| Clone ID | Chr | Pos (cM) | Position in the wheat genome (IWGSC) |
| --- | --- | --- | --- |
| 2265279 | 4D | 1.473 | 4D:498380828-498380903 |
| 1057829 | 4D | 1.496 | 4D:499594538-499594613 |
| 1378820 | 4D | 1.539 | 4D:501675353-501675428 |
| 2249425 | 4D | 1.539 | 4D:500037769-500037826 |
| 1114200 | 4D | 1.571 | 4D:502399154-502399229 |
| 1103052 | 4D | 1.574 | 4D:503617397-503617472 |
| 1101835 | 4D | 1.574 | 4D:502576904-502576979 |
