# Supplementary figures and images for: GWAS revealed a novel resistance locus on chromosome 4D for the quarantine disease Karnal bunt in diverse wheat pre-breeding germplasm
Source: Sci Rep. 2020 Apr 7;10:5999. doi: 10.1038/s41598-020-62711-7 (PMC7138846; doi:10.1038/s41598-020-62711-7)

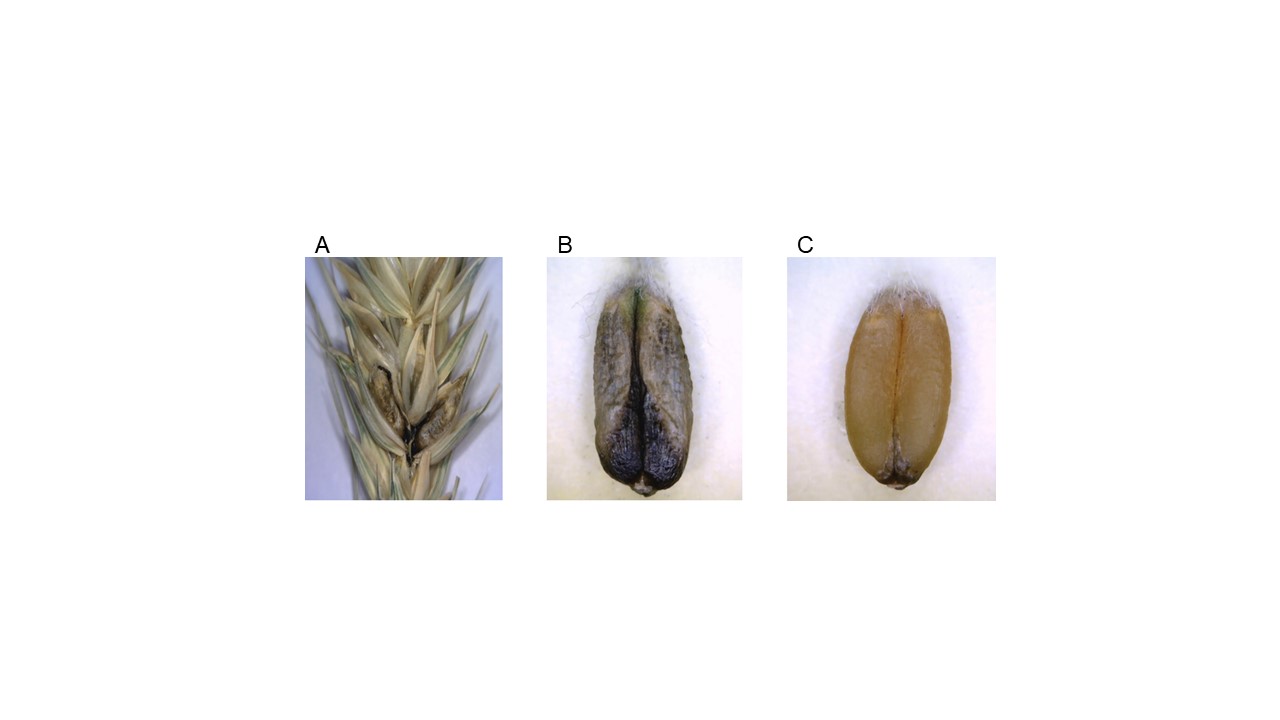

Supplement: Supplementary file 10 — Supplementary material 10. [file 41598_2020_62711_MOESM10_ESM.jpg]

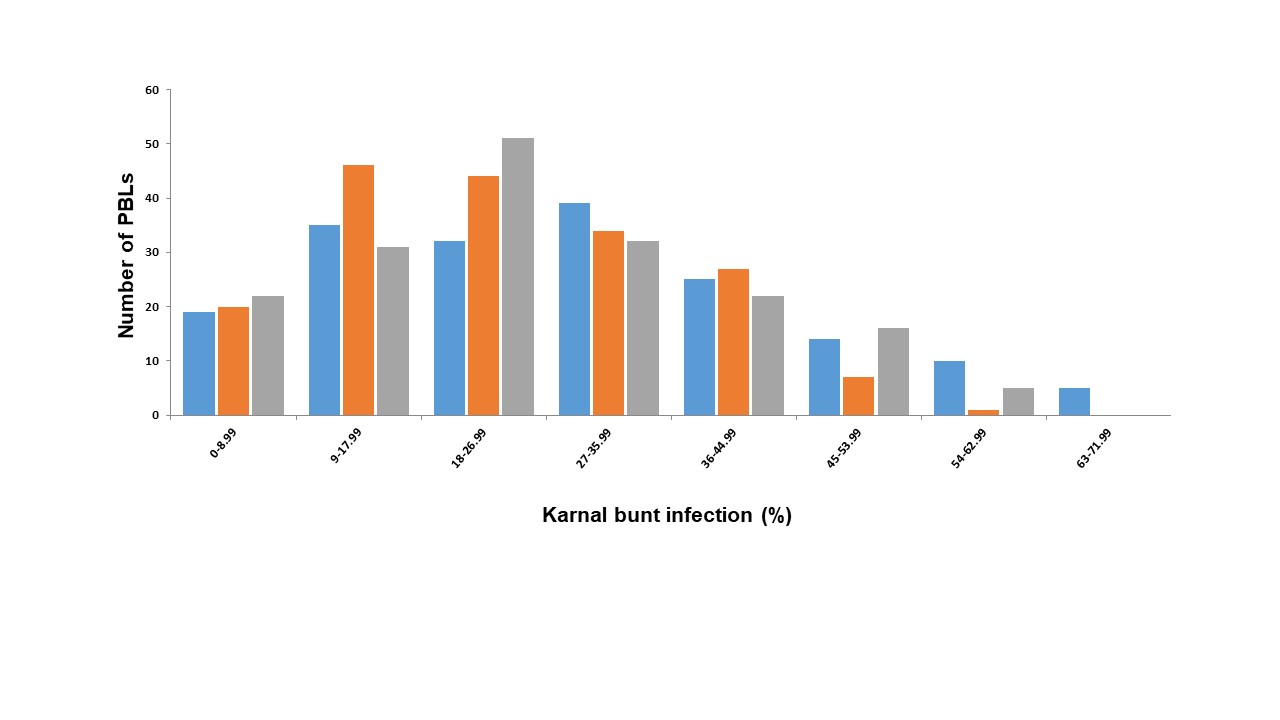

Supplement: Supplementary file 11 — Supplementary material 11. [file 41598_2020_62711_MOESM11_ESM.jpg]

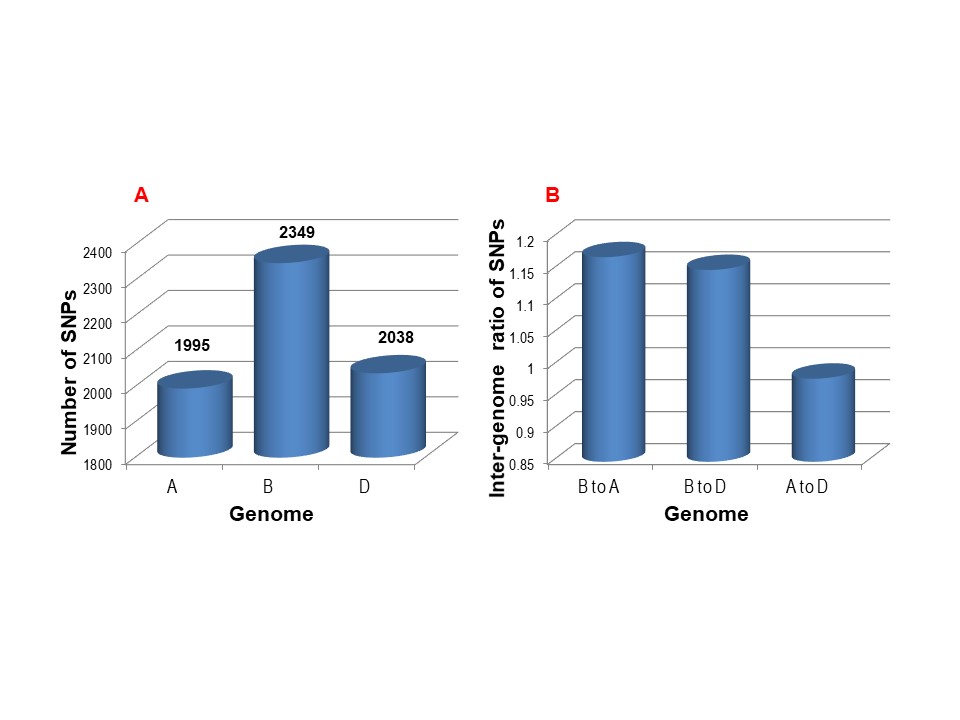

Supplement: Supplementary file 12 — Supplementary material 12. [file 41598_2020_62711_MOESM12_ESM.jpg]

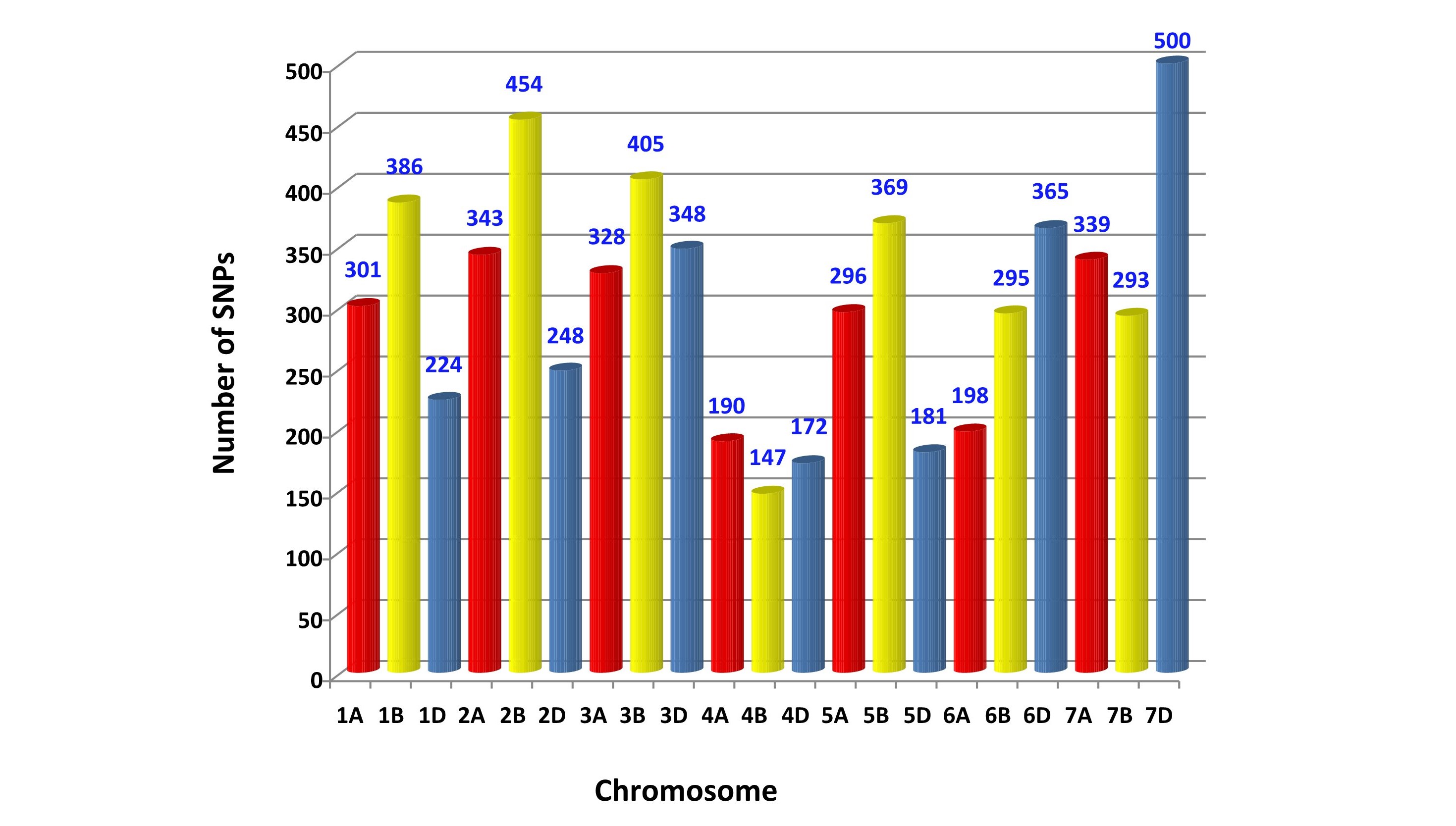

Supplement: Supplementary file 13 — Supplementary material 13. [file 41598_2020_62711_MOESM13_ESM.jpg]

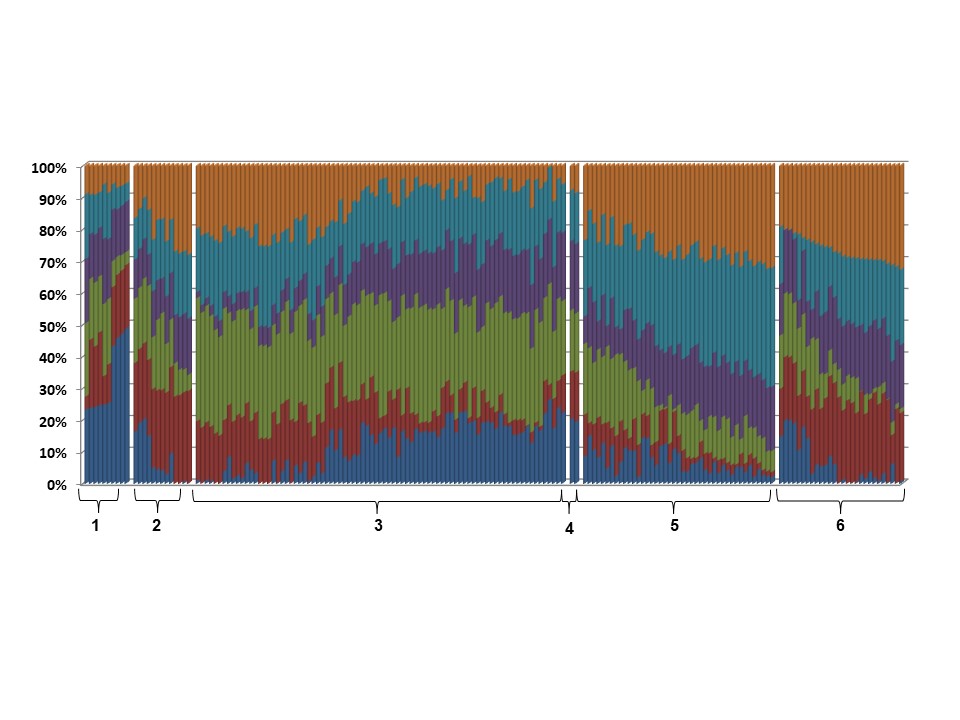

Supplement: Supplementary file 14 — Supplementary material 14. [file 41598_2020_62711_MOESM14_ESM.jpg]

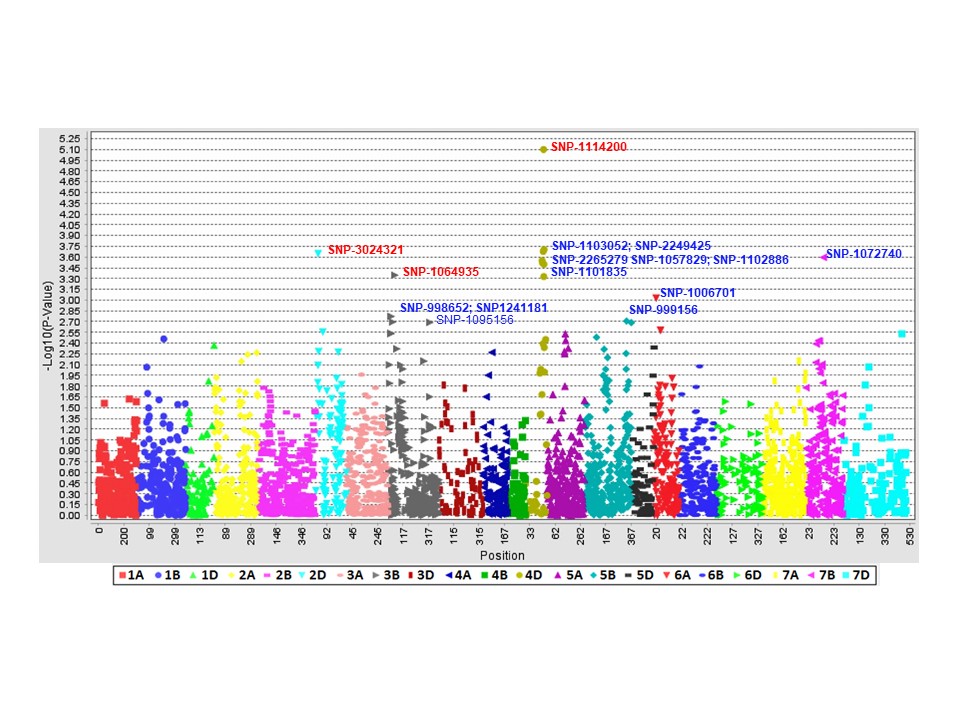

Supplement: Supplementary file 15 — Supplementary material 15. [file 41598_2020_62711_MOESM15_ESM.jpg]

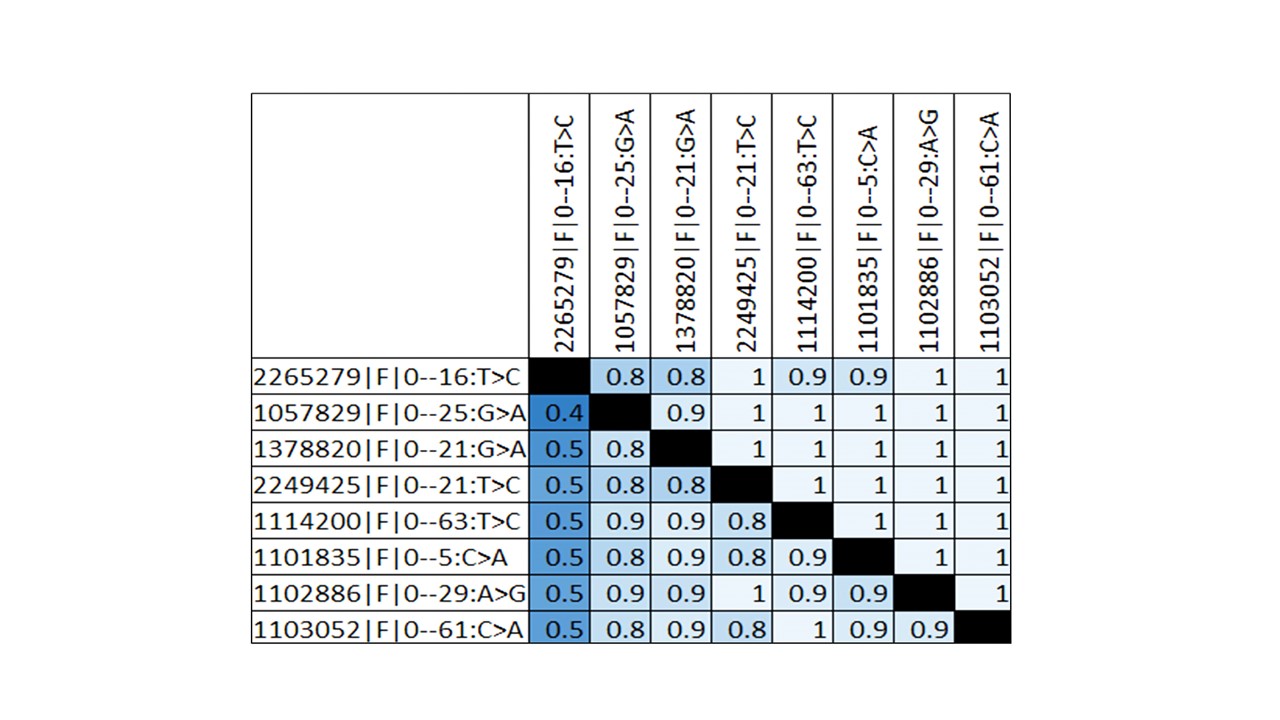

Supplement: Supplementary file 16 — Supplementary material 16. [file 41598_2020_62711_MOESM16_ESM.jpg]

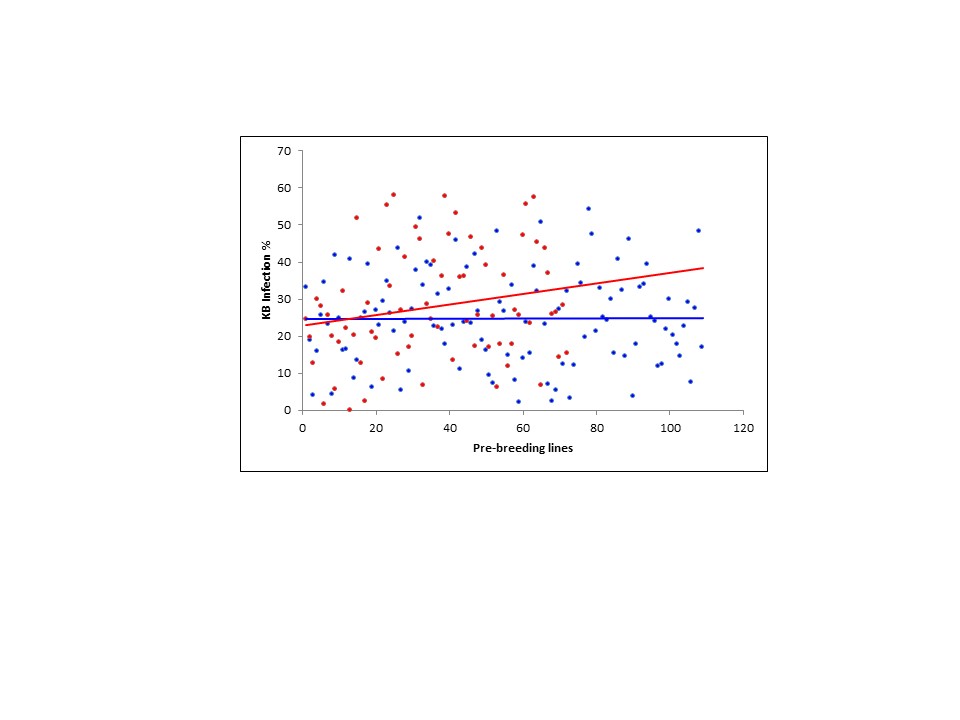

Supplement: Supplementary file 17 — Supplementary material 17. [file 41598_2020_62711_MOESM17_ESM.jpg]

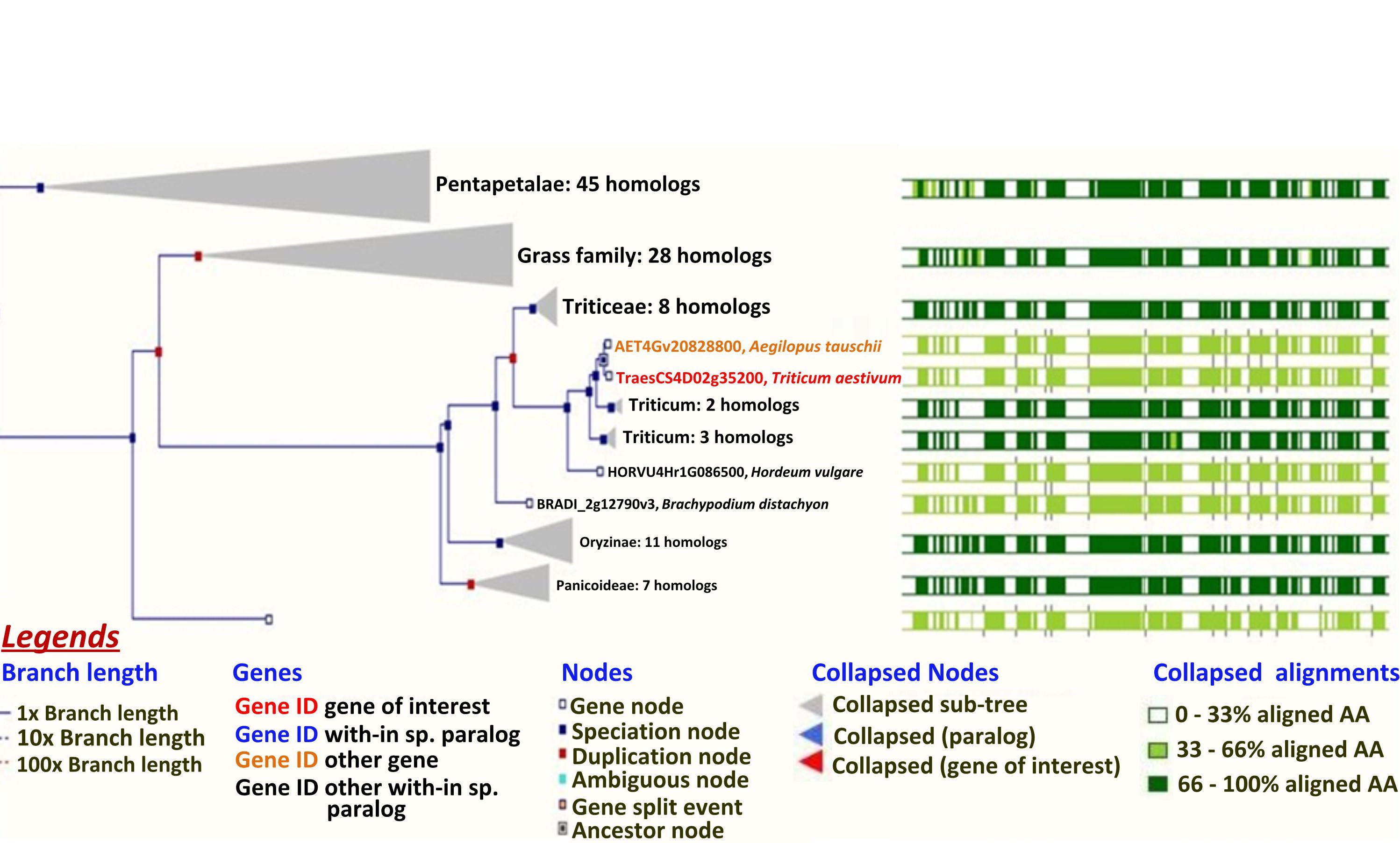

Supplement: Supplementary file 18 — Supplementary material 18. [file 41598_2020_62711_MOESM18_ESM.jpg]

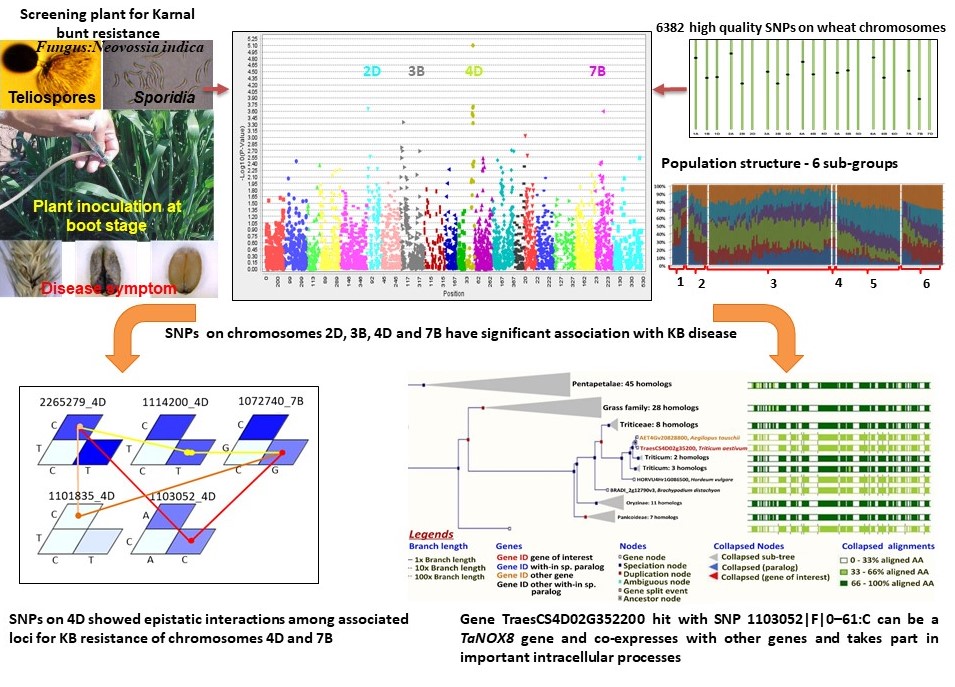

Supplement: Supplementary file 19 — Supplementary material 19. [file 41598_2020_62711_MOESM19_ESM.jpg]
