# Supplementary material for: GWAS revealed a novel resistance locus on chromosome 4D for the quarantine disease Karnal bunt in diverse wheat pre-breeding germplasm
Source: Sci Rep. 2020 Apr 7;10:5999. doi: 10.1038/s41598-020-62711-7 (PMC7138846; doi:10.1038/s41598-020-62711-7)
Supplement: Supplementary file 20 — figure legends [file 41598_2020_62711_MOESM20_ESM.docx]

**Supplementary Figure legends**

**Fig. S1:** A representation of Karnal Bunt disease in (a) an infected spike, (b) fully infected grain, and (c) partially infected grain

**Fig. S2:** Frequency distribution of genotypes based on the severity of Karnal bunt. The X-axis represents the KB infection (%) and the Y-axis represents the number of genotypes. Blue bar = Environment-E1, red bar = Environment-E2, and grey bar = pooled data.

**Fig. S3:** Figure presenting the distribution of markers on three different genomes; (A) distribution of polymorphic DArT markers in wheat genome; X-axis- name of genome and Y-axis-number of markers, (B) ratio of SNP marker between three genomes of wheat; X-axis- comparison between genome and Y-axis-ratio of markers between genome

**Fig. S4:** Chromosome wise distribution of polymorphic DArT-SNP markers in 179 PBLs; X-axis = chromosome name and Y-axis = number of markers; (Red bar = A genome, Yellow bar= B genome, Blue bar = D genome)

**Fig. S5:** Population structure of 179 wheat PBLs at K = 6 based on 6382 polymorphic SNPs.

**Fig. S6:** Genome-wide scan (GWAS analysis) for SNP markers associated with Karnal bunt resistance in 179 PBLs in joint analysis. The plots show SNP based Manhattan plot with the name of most significant SNPs. the chromosomes on the X-axis and the genome-wide scan –log10 (P-values) on the Y-axis

**Fig. S7:** Linkage disequilibrium (LD) across the genomic region on chromosome 4D associated with KB. Numerical LD is presented by blue scale. The pairwise figure between each SNP by both D*j* (upper right) and the more stringent measure r^2^ (bottom left) is presented. The lighter the shading indicates a greater extent of LD between the SNPs

**Fig. S8:** KB Infection score of PBLs with respect to the seven markers on chromosome 4D found significantly associated with the trait. Two groups were formed: (1) Group 1 carrying 109 PBLs with all favorable alleles for 4D markers (represented by blue dots) and Group 2 carrying 70 PBLs with at least 1 unfavorable allele for 4D markers (represented by orange dots). Straight blue trend line of group 1 indicates that allele profile of group 1 PBLs (mean KB % = 24.77±12.51%) is associated with resistance against KB whereas the orange trend line is skewed upwards indicating higher KB infection% (mean KB% = 28.20±15.25%) of group 2 PBLs

**Fig. S9:** Phylogenetic tree of orthologues of TraesCS4D02G352200.

**Fig. S10:** Graphical representation of genetic analysis of diverse pre-breeding lines for Karnal

bunt disease of wheat
